# Efficacy and Safety of Disitamab Vedotin in Patients With Human Epidermal Growth Factor Receptor 2–Positive Locally Advanced or Metastatic Urothelial Carcinoma: A Combined Analysis of Two Phase II Clinical Trials

**DOI:** 10.1200/JCO.22.02912

**Published:** 2023-11-21

**Authors:** Xinan Sheng, Lin Wang, Zhisong He, Yanxia Shi, Hong Luo, Weiqing Han, Xin Yao, Benkang Shi, Jiyan Liu, Changlu Hu, Ziling Liu, Hongqian Guo, Guohua Yu, Zhigang Ji, Jianming Ying, Yun Ling, Shiying Yu, Yi Hu, Jianming Guo, Jianmin Fang, Aiping Zhou, Jun Guo

**Affiliations:** ^1^ Department of Genitourinary Oncology, Key Laboratory of Carcinogenesis and Translational Research (Ministry of Education/Beijing), Peking University Cancer Hospital & Institute, Beijing, China; ^2^ Department of Medical Oncology, National Cancer Center/National Clinical Research Center for Cancer/Cancer Hospital, Chinese Academy of Medical Sciences and Peking Union Medical College, Beijing, China; ^3^ Department of Urology, Peking University First Hospital, Institute of Urology, National Urological Cancer Center of China, Peking University, Beijing, China; ^4^ Department of Medical Oncology, State Key Laboratory of Oncology in South China, Collaborative Innovation Center for Cancer Medicine, Sun Yat-sen University Cancer Center, Guangzhou, China; ^5^ Department of Genitourinary Oncology, Chongqing University Cancer Hospital and Chongqing Cancer Institute and Chongqing Cancer Hospital, Chongqing, China; ^6^ Department of Urology, Hunan Cancer Hospital/The Affiliated Cancer Hospital of Xiangya School of Medicine of Central South University, Changsha, China; ^7^ Department of Genitourinary Oncology, Tianjin Medical University Cancer Institute and Hospital, National Clinical Research Center for Cancer, Tianjin Key Laboratory of Cancer Prevention and Therapy, Tianjin's Clinical Research Center for Cancer, Tianjin, China; ^8^ Department of Urology, Qilu Hospital of Shandong University, Jinan, China; ^9^ Department of Biotherapy, Cancer Center, and National Clinical Research Center for Geriatrics, West China Hospital of Sichuan University, Chengdu, China; ^10^ Department of Medical Oncology, Anhui Provincial Cancer Hospital, Hefei, China; ^11^ Department of Cancer Centre, First Hospital of Jilin University, Changchun, China; ^12^ Department of Urology, Nanjing Drum Tower Hospital, Nanjing, China; ^13^ Department of Medical Oncology, Weifang People's Hospital, Weifang, China; ^14^ Department of Urology, Chinese Academy of Medical Sciences and Peking Union Medical College, Beijing, China; ^15^ Department of Pathology, National Cancer Center/National Clinical Research Center for Cancer /Cancer Hospital, Chinese Academy of Medical Sciences and Peking Union Medical College, Beijing, China; ^16^ Department of Urology, Tongji Hospital, Tongji Medical College of Huazhong University of Science and Technology, Wuhan, China; ^17^ Department of Medical Oncology, Chinese PLA General Hospital, Beijing, China; ^18^ Department of Urology, Zhongshan Hospital, Fudan University, Shanghai, China; ^19^ RemeGen, Ltd, Yantai, China; ^20^ School of Life Science and Technology, Tongji University, Shanghai, China

## Abstract

**PURPOSE:**

To evaluate the efficacy and safety of disitamab vedotin (DV, RC48-ADC), a novel humanized anti–human epidermal growth factor receptor 2 (HER2) antibody conjugated with monomethyl auristatin E, in patients with HER2-positive locally advanced or metastatic urothelial carcinoma (UC) refractory to standard or regular therapies.

**PATIENTS AND METHODS:**

The data analyzed and reported are from two phase II, open-label, multicenter, single-arm studies (RC48-C005 and RC48-C009) in patients with HER2-positive (immunohistochemistry 3+ or 2+) locally advanced or metastatic UC who have progressed on at least one previous line of systemic chemotherapy. Patients received DV treatment (2 mg/kg IV infusion, once every 2 weeks). The primary end point was objective response rate (ORR) assessed by a blinded independent review committee (BIRC). Progression-free survival (PFS), overall survival (OS), and safety were also assessed.

**RESULTS:**

One hundred and seven patients were enrolled in total. The overall confirmed ORR by BIRC was 50.5% (95% CI, 40.6 to 60.3). Consistent results were observed in prespecified subgroups including patients with liver metastasis and patients previously treated with anti–PD-1/L1 therapies. By the cutoff date of May 10, 2022, the median duration of response was 7.3 months (95% CI, 5.7 to 10.8). The median PFS and OS were 5.9 months (95% CI, 4.3 to 7.2) and 14.2 months (95% CI, 9.7 to 18.8), respectively. The most common treatment-related adverse events (TRAEs) were peripheral sensory neuropathy (68.2%), leukopenia (50.5%), AST increased (42.1%), and neutropenia (42.1%). Fifty-eight (54.2%) patients experienced grade ≥3 TRAEs, including peripheral sensory neuropathy (18.7%) and neutropenia (12.1%).

**CONCLUSION:**

DV demonstrated a promising efficacy with a manageable safety profile in patients with HER2-positive locally advanced or metastatic UC who had progressed on at least one line of systemic chemotherapy.

## INTRODUCTION

Locally advanced or metastatic urothelial carcinoma (UC) has a poor prognosis, with only about 5% of patients at stage IV (metastatic) surviving longer than 5 years.^1,2^ Platinum-containing chemotherapy remains the first-line standard of care for advanced UC with the objective response rates (ORRs) of 44.6%-72% historically and the median overall survival (mOS) of 14.0-15.2 months.^2-4^ Recently, multiple immune checkpoint inhibitors, fibroblast growth factor receptor inhibitor (erdafitinib) and several antibody-drug conjugates (ADCs) including enfortumab vedotin (EV), sacituzumab govitecan (SG), and disitamab vedotin (DV, RC48), were approved in platinum-refractory settings in different regions of the world,^3,5-8^ and avelumab was approved as a maintenance treatment after at least disease control after first-line platinum-containing chemotherapy.^3,9^

**CONTEXT**

**Key Objective**
Are disitamab vedotin (a novel humanized anti–human epidermal growth factor receptor 2 [HER2] antibody)-drug conjugates effective and safe for patients with HER2-positive metastatic urothelial carcinoma refractory to standard therapies?
**Knowledge Generated**
More than half of patients (50.5%) achieved objective response with the median progression-free survival (PFS) of 5.9 months and the median overall survival (OS) of 14.2 months and the well-tolerated and manageable safety profile. Similar benefits of objective response rate and the median PFS and OS were observed across all the prespecified subgroups, regardless of the location of primary tumor, metastatic organs including liver metastasis, previous treatment of PD-1/PD-L1 inhibitors, and lines of previous chemotherapy.
**Relevance *(M.A. Carducci)***
This report is among the first HER2 directed therapies for metastatic or locally advanced urothelial cancer to demonstrate meaningful clinical activity. These two China based studies have led to approval there and are being evaluated in large international studies.*
  *Relevance section written by *JCO* Associate Editor Michael A. Carducci, MD, FACP, FASCO.

In bladder cancer, human epidermal growth factor receptor 2 (HER2) overexpression generally correlated with tumor progression and poor prognosis,^10-12^ but monoclonal antibodies and tyrosine kinase inhibitors targeting HER2 all failed to show clinical benefits in metastatic UC (mUC),^13-15^ except that afatinib showed some promising results in patients with mUC-harbored HER2 or HER3 genomic alteration,^16,17^ until the emergence of DV, a novel humanized anti-HER2 antibody conjugated with monomethyl auristatin E (MMAE) via a cleavable linker. The RC48-C005 trial (ClinicalTrials.gov identifier: NCT03507166) was initiated as a pivotal study, a phase II study to assess the efficacy and safety of DV in advanced UC that is HER2-positive (immunohistochemistry [IHC] 3+ or 2+). We observed an ORR of 51.2% and a progression-free survival (PFS) of 6.9 months in 43 patients. As recommended by the regulatory authority (National Medical Products Administration [NMPA]) in China, enrollment in the RC48-C005 trial was terminated and another similar study RC48-C009 trial (ClinicalTrials.gov identifier: NCT03809013) was conducted to go further to confirm the preliminary outcomes of the RC48-C005. Both studies showed promising efficacy and manageable safety in patients with mUC who had progressed on the previous systematic chemotherapy.^6,18^ In January 2022, DV was approved in China for platinum-refractory patients with metastatic UC on the basis of the RC48-C009 study.

We conducted two sequential studies to investigate the safety and efficacy of a new HER2-ADC in patients with HER2-positive chemotherapy-refractory urothelial cancer, and a combined analysis of the two studies with adaptive trial design endorsed by the regulatory authority in China (NMPA) was required to improve the estimation precision of both efficacy and safety. Here, we present the results of this combined analysis.

## PATIENTS AND METHODS

### Study Design and Patients

RC48-C005 and RC48-C009 studies are two open-label, multicenter phase II clinical trials conducted in China, both aiming to evaluate the effectiveness and safety of RC48-ADC (DV) in patients with unresectable, locally advanced, or metastatic UC who failed the previous chemotherapy.

Eligible patients were between age 18 and 80 years with central laboratory–confirmed, histologically HER2-positive UCs that were unresectable, locally advanced, or metastatic. Patients must progress on at least one line of systemic chemotherapy (in RC48-C009, the patient must have been previously treated with gemcitabine, platinum, and taxanes and more heavily treated than those in RC48-C005). Patients must have at least one measurable lesion according to the RECIST version 1.1, with an Eastern Cooperative Oncology Group performance status of 0 or 1 and an expected survival time exceeding 12 weeks. The inclusion and exclusion criteria are listed in the Protocol (online only).

All patients provided written informed consent before joining the study. The study Protocol was approved by the relevant institutional review board or ethics committee of each study center. The study was conducted in compliance with the Declaration of Helsinki and Good Clinical Practice guidelines.

### Procedures

Eligible patients were treated with DV at 2.0 mg/kg as an intravenous infusion over 30-90 minutes (60 minutes was recommended) once every 2 weeks until disease progression, intolerable toxicity, death, or withdrawal of consent. DV dose is based on calculations using bovine serum albumin (BSA)–based extinction coefficient (EC) implemented in China. Outside of China, DV dose calculation is based on DV-based EC, which is equivalent to 1.07 (BSA-based EC) ÷ 1.41 (DV-based EC) × BSA-based EC dose. Dose was modified and interrupted (up to 28 days) in case of treatment-related adverse events (TRAEs) until these events resolved to grade 0/1 or to baseline. Toxicity was managed with supportive care. Survival follow-up was performed for all patients every 12 weeks after the last treatment dose.

The clinical response was evaluated by a blinded independent review committee (BIRC) according to RECIST version 1.1 at baseline and every 6 weeks, irrespective of dose delays or interruptions, until documented disease progression or death.

All adverse events were monitored and graded according to the Common Terminology Criteria for Adverse Events version 4.03. Adverse events are described in preferred terms, as defined in the Medical Dictionary for Regulatory Activities (version 20.1).

### HER2 Testing

These two studies targeted patients with HER2 expression by the IHC staining method. HER2-positive was defined as HER2 IHC 3+ and 2+. IHC scores were reviewed by an independent pathologist at the central laboratory. The detailed IHC methods and representative images of the HER2 IHC grading were previously described.^6^ Gene amplification of *HER2* was also evaluated by fluorescence in situ hybridization (FISH). The staining scores and FISH status were assessed on the basis of the HER2 test guidelines for breast cancer.^19^

### End Points

The primary end point of the two studies was confirmed ORR assessed by BIRC. A confirmed response was defined as complete or partial response demonstrated via computed tomography or magnetic resonance imaging according to RECIST 1.1. The secondary end points included PFS, disease control rate (DCR), duration of response (DoR), OS, and safety.

### Statistical Analysis

The statistical analysis methods of the two studies (RC48-C005 and RC48-C009) were consistent in design, and we maintained close communications and received endorsement from NMPA in China. The study designs including the statistical methods of both RC48-C005 and RC48-C009 are summarized in Appendix Tables A1 and A2 (online only), as well as their differences in the designs (refer to Appendix 1).

For each of the studies, the Clopper-Pearson exact binomial test^20^ was conducted to compare the primary end point (ORR) with the prespecified historical control. The primary end point of ORR was met for each individual study. The same exact binomial tests were also used in pooled analysis for ORR, with the historical control rate and rationale as specified in the RC48-C005 trial. Taken the advantage of the pooled data, the estimates of efficacy and safety profiles are more robust and precise than individual ones.

In the RC48-C005 trial, a sample size of 60 allowed a 97% power and a one-sided α level of .025 to reject the null hypothesis with an expected 30% ORR of the investigational drug against an ORR of 10% in control and a dropout rate of 10%. Both ORRs (expected and control) were determined after consultation with NMPA on the basis of the reported range globally in 2016-2017^21^ and expert experience. A total of 43 patients were enrolled and followed up in RC48-C005. The study was stopped early because of high efficacy observed in the prespecified interim analysis. A promising ORR of 66.7% and a DCR of 93.3% were observed from data of the first 30 patients. The early stopping of RC48-C005 was also agreed by NMPA.

In the RC48-C009 trial, the planned sample size of 60 patients allowed a 95% power and a one-sided α level of .025 to detect an ORR of 52% against a control ORR of 30% with a dropout rate of 10%. The ORR of 52% is estimated on the basis of partial follow-up data of RC48-005. The control ORR of 30% was determined after communication with NMPA and considered the highest ORR among existing treatment options^22,23^ and the early readouts of the RC48-C005 study.

Two sensitivity analyses of median PFS (mPFS) were performed. The first sensitivity analysis censored patients at the date of last tumor assessment with documented nonprogression before the start of alternative treatments. The second one censored patients at the date of last tumor assessment with documented nonprogression before more than one missed visit. In addition, the median follow-up was calculated as the interval between the first DV treatment and the last known survival visit among patients censored for OS.

All statistical analyses were conducted using SAS 9.4 (SAS Institute Inc, Cary, NC). The full statistical analysis plan for each of the two studies is presented in Appendix 1.

## RESULTS

**FIG 1.**
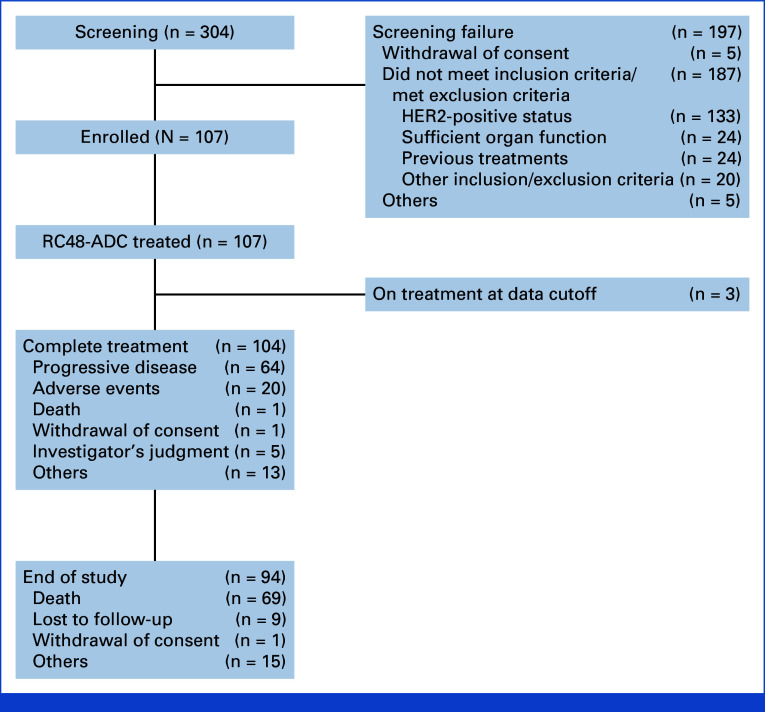
Overall flowchart. ^a^For the C005 study, two patients had two reasons for screening failure. For the C009 study, 12 patients had two reasons for screening failure. HER2, human epidermal growth factor receptor 2.

In the combined analysis, 304 patients were screened and 107 were enrolled from 17 sites (Fig 1). The RC48-C005 trial was conducted on December 28, 2017, and the RC48-C009 trial was conducted on December 19, 2018. The last few patients of RC48-C005 and RC48-C009 were enrolled on November 28, 2018, and September 4, 2020, respectively. The data cutoff date for RC48-C005 is May 6, 2020, whereas the data cutoff date for RC48-C009 is May 10, 2022, which was also the cutoff date of survival follow-up in this combined analysis.

The 107 patients enrolled in the two trials included 80 males (74.8%), with a median age of 63.0 years (range, 40-79). About half of the patients had a primary tumor from the bladder (51.4%), and the others had upper tract UC (UTUC; renal pelvis, 26.2%; ureter, 28.2%). All the patients had metastatic disease. Most of the patients (97 patients, 90.7%) had visceral metastasis, including 48 patients (44.9%) of liver metastasis. Before starting the DV treatment, most patients (69 patients, 64.5%) had received more than one previous systematic treatment and 27 patients (25.2%) had received PD-1/PD-L1 inhibitor treatment. On the basis of the central laboratory test results, the number of patients confirmed with HER2 IHC 2+ and 3+ was 67 (62.6%) and 40 (37.4%), respectively. Five were tested as FISH+ and 53 were tested as FISH– in the patients with IHC HER2 2+, whereas the FISH status of the other nine patients with IHC HER2 2+ was unknown. The baseline characteristics of the two pooled studies' populations are listed in Table 1.

**TABLE 1.** Baseline Demographic and Clinical Characteristics

Patient Characteristic	RC48-C005 (n = 43)	RC48-C009 (n = 64)	Overall (n = 107)	*P* ^a^
Age, years				
Median	64.0	62.5	63.0	
Mean (standard deviation)	62.3 (8.18)	61.5 (8.14)	61.8 (8.13)	.605
Min-max	45-75	40-79	40-79	
Sex, No. (%)				.699
Male	33 (76.7)	47 (73.4)	80 (74.8)	
Female	10 (23.3)	17 (26.6)	27 (25.2)	
Primary lesion, No. (%)				
Bladder	21 (48.8)	34 (53.1)	55 (51.4)	.664
Renal pelvis	14 (32.6)	14 (21.9)	28 (26.2)	.218
Ureter	10 (23.3)	20 (31.3)	30 (28.0)	.367
HER2 status,^b^ No. (%)				.723
IHC 3+ or IHC2+ FISH+^c^	20 (46.5)	25 (39.1)	45 (42.1)	
IHC2+ FISH–	20 (46.5)	33 (51.6)	53 (49.5)	
IHC2+, FISH unknown^d^	3 (7.0)	6 (9.4)	9 (8.4)	
Current extent of disease, No. (%)				
Metastatic	43 (100.0)	64 (100.0)	107 (100.0)	
Metastasis sites, No. (%)	40 (93.0)	63 (98.4)	103 (96.3)	
Lymph nodes	32 (74.4)	54 (84.4)	86 (80.4)	.204
Lungs	22 (51.2)	31 (48.4)	53 (49.5)	.782
Liver	20 (46.5)	28 (43.8)	48 (44.9)	.778
Bone	16 (37.2)	32 (50.0)	48 (44.9)	.192
No. of previous systemic therapies, No. (%)				<.001
Only one line	29 (67.4)	9 (14.1)	38 (35.5)	
Greater than or equal to two lines	14 (32.6)	55 (85.9)	69 (64.5)	
Previous therapies, No. (%)	36 (83.7)	56 (87.5)	92 (86.0)	
Cisplatin-containing chemotherapy	35 (81.4)	51 (79.7)	86 (80.4)	.827
PD-1/PD-L1 therapy	8 (18.6)	19 (29.7)	27 (25.2)	.196
Paclitaxel-containing chemotherapy	15 (34.9)	64 (100.0)	79 (73.8)	<.001
Bellmunt score, No. (%)^e^				.560
0	7 (16.3)	18 (28.1)	25 (23.4)	
1	17 (39.5)	22 (34.4)	39 (36.4)	
2	17 (39.5)	21 (32.8)	38 (35.5)	
3	2 (4.7)	3 (4.7)	5 (4.7)	

Abbreviations: ECOG PS, Eastern Cooperative Oncology Group Performance Status; FISH, fluorescence in situ hybridization; HER2, human epidermal growth factor receptor 2; HGB, hemoglobin; IHC, immunohistochemistry; UC, urothelial carcinoma.

^a^ *P* values for comparison of sex, primary lesion, HER2 status, current extent of disease, metastasis sites, number of previous systemic therapies, previous therapies, and Bellmunt score are based on the chi-square test; *P* value for age comparison is based on the *t* test.

^b^ FISH testing was performed for all the patients with IHC 2+.

^c^ Forty patients (37.4%) were classified as IHC 3+, and five patients (4.7%) as IHC2+ FISH+.

^d^ The FISH testing results for nine patients are unknown.

^e^ The Bellmunt risk score categorizes patients into four risk groups by assigning a point for each of three risk factors; an ECOG PS of >0, a HGB level of <10 g/dL, and the presence of liver metastases.

**FIG 2.**
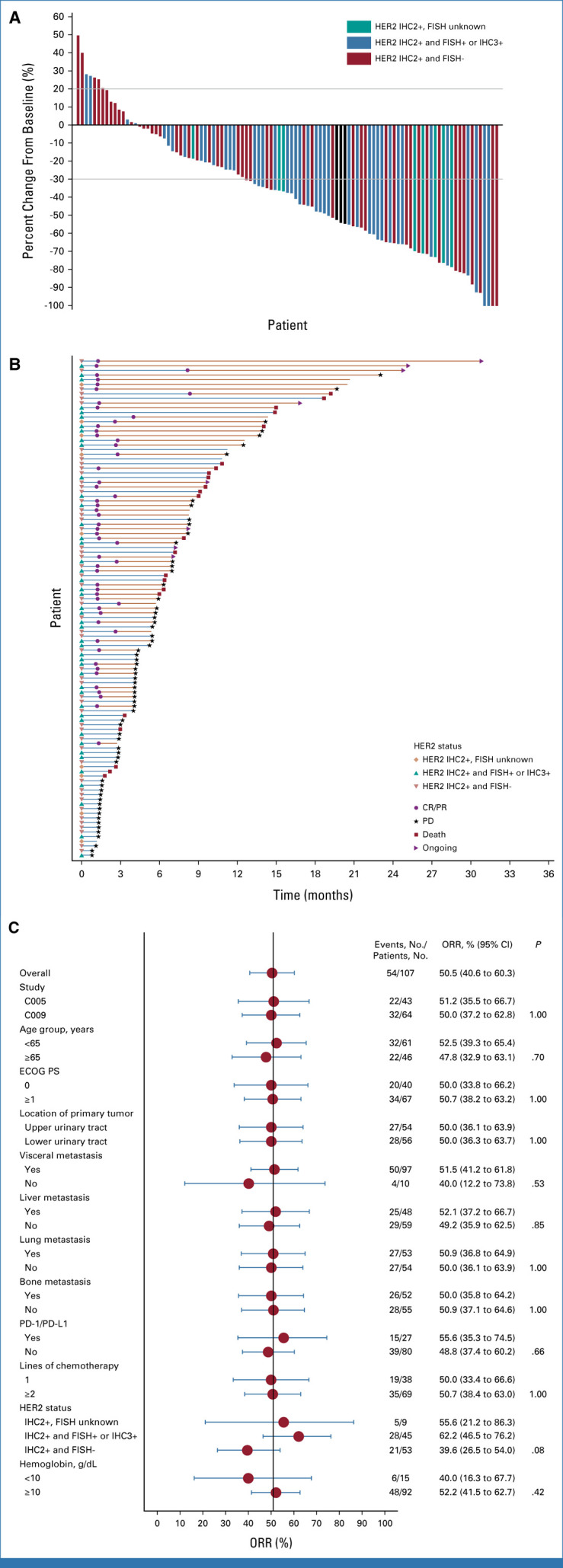
(A) Waterfall plot of the best percent change from baseline in the sum of the diameters of target lesions. Eighty-eight (82.2%) patients had a decrease in tumor size from baseline as assessed by the BIRC. (B) Swimmer plot of patients' responses from the start of treatment to PD assessed by the BIRC, death, or withdrawal. Fifty-four patients (50.5%) were responders whose target lesion decreased by not <30%, and one patient (0.9%) and 53 patients (49.5%) were assessed as confirmed CR and PR, respectively. The best response of 34 patients (31.8%) was SD, and 19 patients had PD. (C) Forest plot of the ORRs in key subgroups. The subgroups were based on the baseline disease characteristics. The confirmed ORR was evaluated in the key prespecified subgroups per the BIRC. BIRC, blinded independent review committee; CR, complete response; ECOG PS, Eastern Cooperative Oncology Group Performance Status; FISH, fluorescence in situ hybridization; HER2, human epidermal growth factor receptor 2; IHC, immunohistochemistry; ORR, objective response rate; PD, progressive disease; PR, partial response; SD, stable disease.

As assessed by the BIRC (Table 2), 54 patients (50.5%; 95% CI, 40.6 to 60.3) had confirmed objective responses according to RECIST 1.1, including two (1.9%) complete responders and 52 (48.6%) partial responders (PR). In addition, 34 patients (31.8%) experienced stable disease (SD) as the best response. The decrease in target lesions from baseline was observed in 88 (82.2%) of these patients (Fig 2A). The DCR was 82.2% (95% CI, 73.7 to 89.0; Table 2). The median DoR was 7.3 months (95% CI, 5.7 to 10.8; Table 2; Fig 2B).

**TABLE 2.** Tumor Responses as Assessed by the BIRC

End Point	RC48-C005 (n = 43)	RC48-C009 (n = 64)	Overall (n = 107)
Confirmed objective response rate, % (95% CI)	51.2 (35.5 to 66.7)	50.0 (37.2 to 62.8)	50.5 (40.6 to 60.3)
IHC2+ and FISH+ or IHC3+	60.0 (36.1 to 80.9)	64.0 (42.5 to 82.0)	62.2 (46.5 to 76.2)^a^
IHC2+ and FISH–	40.0 (19.1 to 63.9)	39.4 (22.9 to 57.9)	39.6 (26.5 to 54.0)^a^
IHC2+ and FISH unknown	66.7 (9.4 to 99.2)	50.0 (11.8 to 88.2)	55.6 (21.2 to 86.3)^a^
Best overall response, No. (%) (95% CI)			
Complete response	0 (0.0) (0.2 to 6.6)	2 (3.1) (0.4 to 10.8)	2 (1.9) (0.2 to 6.6)
Partial response	22 (51.2) (38.8 to 58.5)	30 (46.9) (34.3 to 59.8)	52 (48.6) (38.8 to 58.5)
Stable disease	17 (39.5) (23.1 to 41.5)	17 (26.6) (16.3 to 39.1)	34 (31.8) (23.1 to 41.5)
Progressive disease	3 (7.0) (8.1 to 22.1)	12 (18.8) (10.1 to 30.5)	15 (14.0) (8.1 to 22.1)
Not evaluable	1 (2.3) (1.0 to 9.3)	3 (4.7) (1.0 to 13.1)	4 (3.7) (1.0 to 9.3)
DCR, No. (%) (95% CI)	39 (90.7) (77.9 to 97.4)	49 (76.6) (64.3 to 86.2)	88 (82.2) (73.7 to 89.0)
Duration of response, months (95% CI)	7.0 (4.7 to 12.4)	8.3 (4.3 to 12.6)	7.3 (5.7 to 10.8)
PFS			
Median, months (95% CI)	6.9 (5.4 to 9.0)	5.3 (4.0 to 7.2)	5.9 (4.3 to 7.2)
12-month rate, % (95% CI)	23.6 (11.5 to 38.1)	25.3 (15.0 to 36.9)	24.7 (16.5 to 33.7)
OS			
Median, months (95% CI)	13.9 (9.1 to NE)	14.8 (8.7 to 18.6)	14.2 (9.7 to 18.8)
18-month rate, % (95% CI)	46.3 (30.9 to 60.3)	39.6 (27.2 to 51.6)	42.2 (32.5 to 51.5)
OS follow-up, months, median	19.6	23.4	20.5

Abbreviations: BIRC, blinded independent review committee; DCR, disease control rate; FISH, fluorescence in situ hybridization; IHC, immunohistochemistry; NE, not estimable; OS, overall survival; PFS, progression-free survival.

^a^ There is no statistical difference among the three subgroups for C005, C009, and overall with *P* = .4441, *P* = .1649, and *P* = .0798, respectively.

Subgroup analyses (Fig 2C) indicated that the ORR was 50.0% in the 38 patients with only one previous line of chemotherapy and 50.7% in the 69 patients with two or more lines of treatment. Among 48 patients with liver metastasis, the confirmed ORR was 52.1%. Among the 27 patients who have received previous PD-1/PD-L1 inhibitors, the confirmed ORR was 55.6%. The ORR in patients with UTUC or bladder UC was both 50.0%. The ORR was numerically higher for patients with higher HER2 expression (defined as either HER2 IHC 2+ and FISH-positive or IHC 3+; ORR, 62.2%) than patients with lower HER2 expression (defined as HER2 IHC 2+ and FISH-negative; ORR, 39.6%). Of the nine patients with IHC 2+ and unknown FISH results, five patients had partial response (ORR, 55.6%). However, no statistical significance was seen.

**FIG A2.**
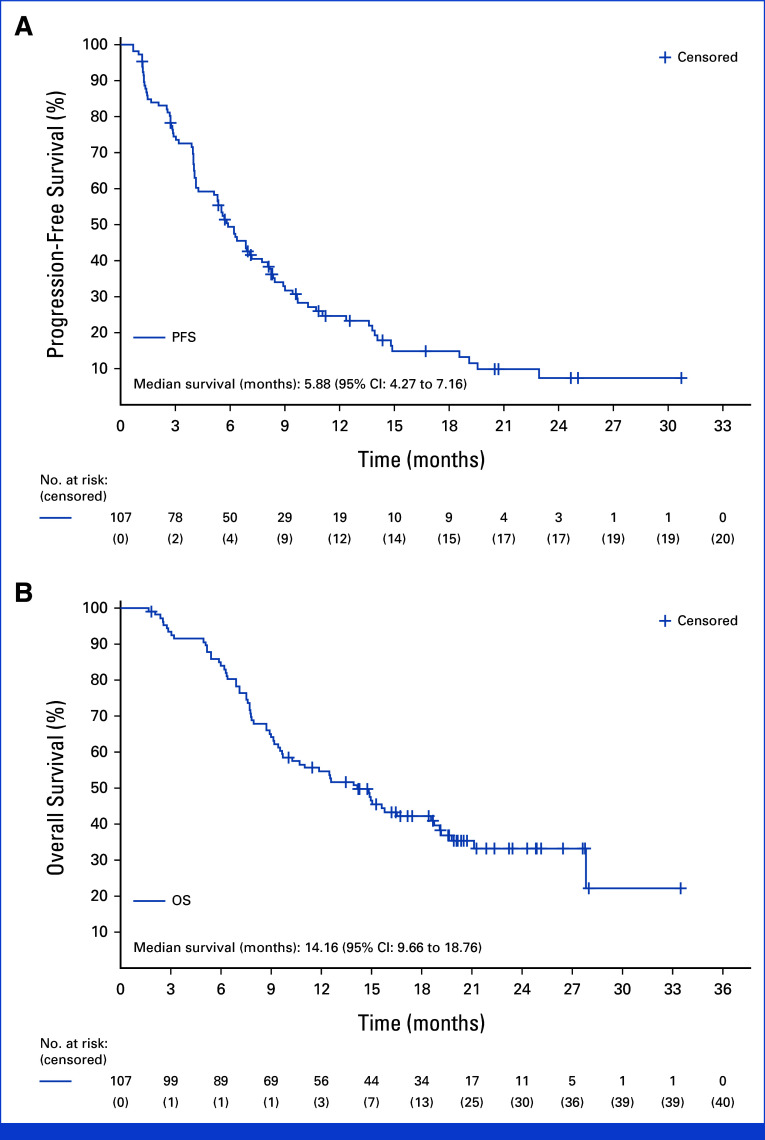
Kaplan-Meier estimates of (A) PFS as assessed by the BIRC and (B) OS. BIRC, blinded independent review committee; OS, overall survival; PFS, progression-free survival.

**FIG A3.**
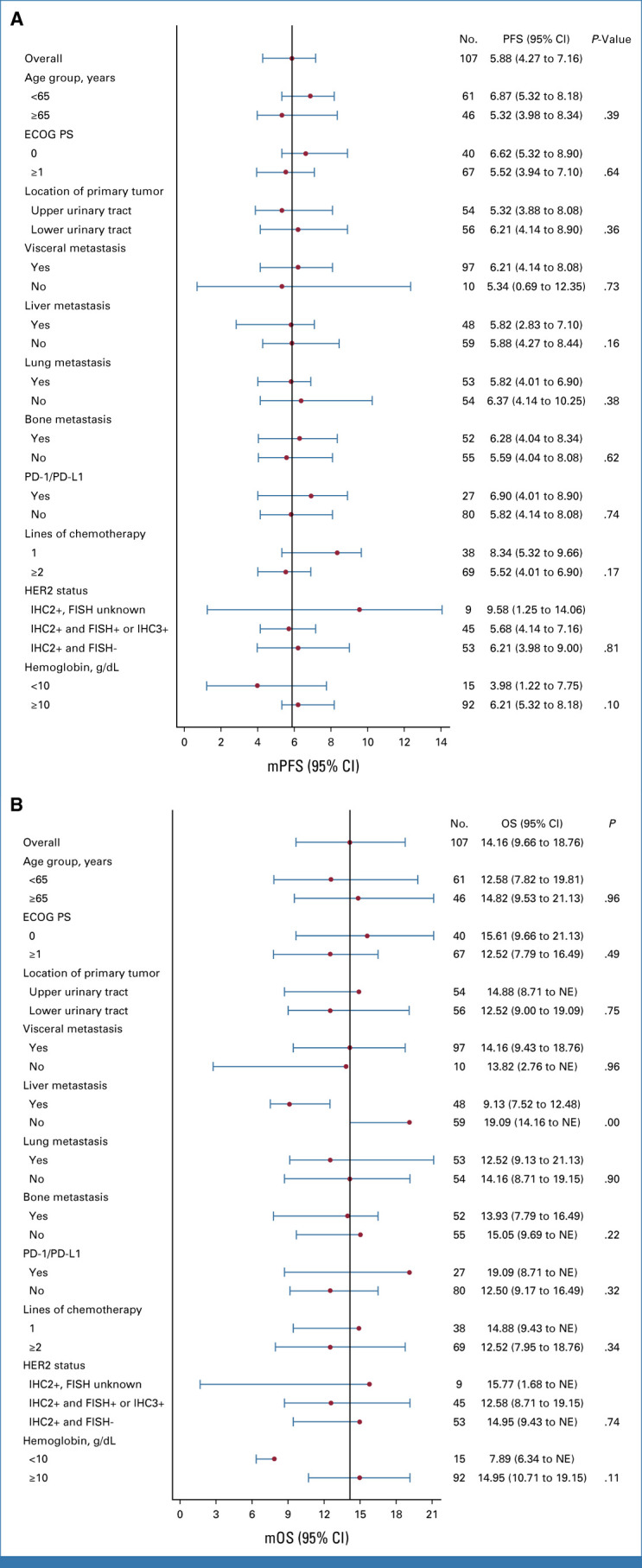
Forest plot of the (A) mPFS and (B) mOS in key subgroups. The subgroups were based on the baseline disease characteristics. ECOG PS, Eastern Cooperative Oncology Group Performance Status; FISH, fluorescence in situ hybridization; HER2, human epidermal growth factor receptor 2; IHC, immunohistochemistry; mOS, median OS; mPFS, median PFS; NE, not estimable; OS, overall survival; PFS, progression-free survival.

By the cutoff date of May 10, 2022, the median follow-up (from the first dose to the data cutoff date) was 20.5 months. The mPFS was 5.9 (95% CI, 4.3 to 7.2) months, with a 12-month PFS rate of 24.7% (95% CI, 16.5 to 33.7; Table 2; Appendix Fig A2A). According to the sensitivity analyses, the overall mPFS was 5.8 (95% CI, 4.3 to 6.9) months. The mOS was 14.2 (95% CI, 9.7 to 18.8) months, and the 18-month OS rate was 42.2% (95% CI, 32.5 to 51.5; Table 2; Appendix Fig A2B). Subgroup analyses indicated that the mPFS and mOS had no significant difference across all subgroups, including the location of primary tumor, visceral metastases, previous chemotherapy, previous treatment of PD-1/PD-L1 inhibitors, and HER2 status (Appendix Figs A3A and A3B).

The median treatment duration for RC48-ADC was 21.3 weeks (range, 2.0-143.4). The median dose intensity was 96.0% (range, 40.0%-100.0%). In this study, the median time to treatment discontinuation for treatment-related reasons is 154 days. Nineteen patients discontinued treatment owing to grade 2 (n = 4) and grade 3 (n = 15) TRAEs, in whom the proportion of patients with PR and SD was 68.4% (13 of 19) and 31.6% (6 of 19), respectively. Furthermore, 32 patients had a dose reduction because of TRAE and only one patient discontinued treatment, implying that dose reduction was effective in avoiding permanent discontinuation of study treatment. The most reported TRAE resulting in drug discontinuation was peripheral sensory neuropathy (12, 11.2%). As assessed by the BIRC, all 107 patients experienced at least one TRAE (Table 3). The most reported TRAEs were peripheral sensory neuropathy (68.2%), leukopenia (50.5%), neutropenia (42.1%), AST increased (42.1%), alopecia (40.2%), asthenia (39.3%), ALT increased (35.5%), and decreased appetite (31.8%; Table 3). No grade 4 or 5 TRAEs were observed. Fifty-eight (54.2%) patients experienced grade 3 TRAEs. The most reported grade 3 TRAEs were peripheral sensory neuropathy (18.7%) and neutropenia (12.1%).

**TABLE 3.** TRAEs (≥10%) in All Patients

TRAE	Grade 1, No. (%)	Grade 2, No. (%)	Grade 3, No. (%)	Grade 4, No. (%)	Grade 5, No. (%)	Total (n = 107), n (%)
Any TRAE	13 (12.1)	36 (33.6)	55 (51.4)	3 (2.8)	0	107 (100.0)
Peripheral sensory neuropathy	35 (32.7)	18 (16.8)	20 (18.7)	0	0	73 (68.2)
Leukopenia	19 (17.8)	33 (30.8)	2 (1.9)	0	0	54 (50.5)
AST increased	39 (36.4)	5 (4.7)	1 (0.9)	0	0	45 (42.1)
Neutropenia	13 (12.1)	19 (17.8)	12 (11.2)	1 (0.9)	0	45 (42.1)
Alopecia	37 (34.6)	5 (4.7)	1 (0.9)	0	0	43 (40.2)
Asthenia	26 (24.3)	12 (11.2)	4 (3.7)	0	0	42 (39.3)
ALT increased	31 (29.0)	7 (6.5)	0	0	0	38 (35.5)
Decreased appetite	30 (28.0)	3 (2.8)	1 (0.9)	0	0	34 (31.8)
Nausea	27 (25.2)	4 (3.7)	0	0	0	31 (29.0)
Weight decreased	16 (15.0)	11 (10.3)	0	0	0	27 (25.2)
Platelet count decreased	14 (13.1)	12 (11.2)	0	0	0	26 (24.3)
Constipation	21 (19.6)	3 (2.8)	0	0	0	24 (22.4)
Blood triglycerides increased	17 (15.9)	5 (4.7)	1 (0.9)	1 (0.9)	0	24 (22.4)
Anemia	12 (11.2)	8 (7.5)	3 (2.8)	0	0	23 (21.5)
Gamma-glutamyltransferase increased	7 (6.5)	9 (8.4)	6 (5.6)	0	0	22 (20.6)
Pruritus	13 (12.1)	7 (6.5)	1 (0.9)	0	0	21 (19.6)
Vomiting	16 (15.0)	2 (1.9)	1 (0.9)	0	0	19 (17.8)
Blood creatine phosphokinase increased	11 (10.3)	2 (1.9)	2 (1.9)	1 (0.9)	0	16 (15.0)
Blood glucose increased	8 (7.5)	6 (5.6)	2 (1.9)	0	0	16 (15.0)
Hemoglobin decreased	7 (6.5)	5 (4.7)	1 (0.9)	0	0	13 (12.1)
Protein urine present	4 (3.7)	7 (6.5)	1 (0.9)	0	0	12 (11.2)
Rash	9 (8.4)	3 (2.8)	0	0	0	12 (11.2)
Pyrexia	6 (5.6)	5 (4.7)	0	0	0	11 (10.3)
Pain in extremity	8 (7.5)	3 (2.8)	0	0	0	11 (10.3)

Abbreviation: TRAE, treatment-related adverse event.

## DISCUSSION

To our knowledge, the RC48-C005 study is the first phase II trial of evaluating HER2-targeting ADC (DV) in HER2 IHC 3+ or 2+ patients with mUC who have progressed on at least one previous line of chemotherapy, which demonstrated promising efficacy (ORR, 51.2%; mPFS, 6.9 months; mOS, 13.9 months) with a manageable safety profile. The combined analysis of RC48-C005 and RC48-C009 demonstrated the consistently promising efficacy of DV in HER2-positive, chemotherapy-refractory patients with mUC, with an overall ORR of 50.5%, a PFS of 5.9 months, and an OS of 14.2 months. The treatment effects were consistent in all key subgroups.

Asian patients with mUC carried a characteristic of a higher incidence rate of UTUC than the Western population, generally about 20%-36% in most Asian cohorts with mUC.^24,25^ Most of the mUC studies in the standard therapy failure settings observed a relatively better efficacy in lower tract UC (LTUC) than UTUC.^5,9,26^ In the current study, the ORRs of patients with both UTUC and LTUC were 50.0% (Fig 2C) and the mPFS (5.3 months *v* 6.2 months) and mOS (14.9 months *v* 15.2 months) of UTUC were comparable with those of LTUC, which suggested that DV has comparable performance in patients with metastatic UTUC to LTUC.

As indicated in the current study, HER2-targeting DV achieved favorable outcomes in patients with HER2-positive advanced UC, with an ORR of 62.2% in the IHC2+ and FISH+ or IHC3+ patients. The phase III trial with a larger sample size with adequately represented HER2 expression subgroups could help to address this issue. In addition, we also observed a moderate ORR in IHC 2+ and FISH-negative patients (39.6%). This provides a potential of DV treatment in patients with low or even negative HER2 expression. Accordingly, the RC48-C011 study (ClinicalTrials.gov identifier: NCT04073602) of DV in patients with HER2-negative (IHC 0 or 1+) metastatic UC was developed and completed the enrollment. The preliminary result of the RC48-C011 study showed certain antitumor activity of DV in HER2-negative metastatic UC with a confirmed ORR of 26.3%, a mPFS of 5.6 months, and a mOS of 16.4 months.^27^

EV and SG targets for nectin-4 and trop-2 were approved for use in the unselected patient population. In comparison, DV previously required HER2-positive patients. Given the emerging data from the RC48-C011 study,^27^ HER2 screening might not be needed for DV in the future. Further clinical trials are warranted to test this hypothesis. In fact, except for the RC48-C011 trial, the RC48G001 trial (ClinicalTrials.gov identifier: NCT04879329) was also developed to test DV alone or in combination with pembrolizumab in HER2-positive or HER2-low patients with mUC, whereas the RC48-C014 trial (ClinicalTrials.gov identifier: NCT04264936) is ongoing to test the combination of DV with toripalimab, an anti–PD-1 inhibitor, in mUC without restriction of HER2 expression status.

Early progression on previous platinum-based chemotherapy and a shorter time between the initial time of platinum-based chemotherapy and subsequent therapy were considered to be associated with poor outcomes in mUC.^28^ In the current combined analyses, no differences in the ORRs (53.3% *v* 42.9% *v* 52.2%) and the mPFS (5.4 months *v* 6.2 months *v* 6.2 months) were observed when comparing patients with different median times from initiation of the previous chemotherapy to first DV treatment (<3 months *v* 3-6 months *v* >6 months). Hence, the influence of clinical response to previous chemotherapy on the subsequent DV treatment needs to be further explored in a future study on DV treatment.

Several ADC drugs had been approved for the treatment of mUC after progression on systemic therapy, including EV, SG, and DV.^5-7^ As to those patients with mUC who had progressed on both chemotherapy and anti–PD-1/L1 therapy, the ORRs after the EV treatment ranged from 40.6% to 44.0%, with the mPFS from 5.6 months to 5.8 months and the mOS from 11.7 months to 12.9 months, as indicated in EV201 and EV301 studies.^5,26^ Cohort 1 results from the TROPHY-U-01 study showed that the ORR of SG treatment in the same setting was 27%, with the mPFS and OS being 5.4 months and 10.9 months, respectively.^7^ According to the current study, DV demonstrated a comparable ORR (55.6%), mPFS (6.9 months), and mOS (19.1 months; Appendix Fig A3). Although the comparison among separate clinical trials could be problematic and challenging because of possible selection bias, confounding, and other factors, the efficacy data from the above trials suggested the promising role of DV in patients with mUC who progressed on both chemotherapy and anti–PD-1/L1 therapy.

Given that these ADCs achieved success in the chemotherapy-refractory settings in patients with mUC, the next step is to move forward the use of ADCs to the frontline treatment. Without exception, these ADCs moved forward to choose combination with the PD-1/L1 inhibitors. Both cohort A of the EV-103 trial^29^ and cohort K of the EV-103 trial^30^ used EV in combination with pembrolizumab as the first-line therapy to treat the cisplatin-ineligible patients with mUC and demonstrated the ORRs of 64.5%-73.3%. The median DoR of cohort A in the EV-103 trial was 25.6 months, whereas the median DoR of cohort K in the EV-103 trial was not reached with a median follow-up of 14.8 months. DV was also set to combine with toripalimab to treat the patients with mUC in the RC48-C014 trial (ClinicalTrials.gov identifier: NCT04264936),^31^ and 73.9% of confirmed ORR in first-line therapy was achieved as their preliminary results indicated. These data support EV or DV plus PD-1 inhibitor as the first-line treatment in cisplatin-ineligible patients with mUC. On the basis of these data, the FDA granted accelerated approval to EV with pembrolizumab for cisplatin-ineligible patients with locally advanced or metastatic UC on April 3, 2023,^32^ whereas a phase III EV-302 trial is ongoing to compare EV plus pembrolizumab versus cisplatin-containing chemotherapy in the first-line treatment of patients with cisplatin-eligible mUC (ClinicalTrials.gov identifier: NCT04223856). However, the TROPHY-U-01 trial of cohort 3 attempted to combine SG with pembrolizumab as second-line therapy in checkpoint inhibitor–naive patients with mUC who progressed after platinum-based chemotherapy, and after a median follow-up of 12.5 months, the ORR was 41% and the mPFS was 5.3 months, whereas the median time to response was 1.4 months and the median OS was 12.7 months.^33^ The role of SG plus pembrolizumab in first-line therapy needs to be explored further.

DV-induced toxicity was clinically manageable in the current combined analysis. The most common TRAEs included leukopenia, liver function injury, and peripheral sensory neuropathy. These AEs were considered to be mostly related to the toxicity of DV payload, MMAE. No grade 4 or 5 TRAEs were observed. When compared with the previous reports from the RC48-C005 study, the incidence rate of peripheral sensory neuropathy in this combined study was relatively lower (all grade, 68.2% *v* 74.5%; grade 3, 18.7% *v* 25.6%). Given the larger sample size and longer follow-up than the RC48-C005 study, this combined analysis could eventually reflect the real status of peripheral sensory neuropathy including its incidence rate and grade after the treatment of DV.

One of the limitations of this study is that the enrollment was exclusively in China, while its applicability to Western populations remains unknown. To explore the efficacy and safety of DV treatment in Western populations, an international, multicenter, multicohort, phase II study was developed (RC48G001 study; ClinicalTrials.gov identifier: NCT04879329), including sites from North America, Europe, Latin America, Asia-Pacific, and Israel. Because of the limitation of the nonrandomized trial including the possible selection biases and heterogeneity of mixing two trial populations, there is a need to evaluate the DV in a broader population of patients with metastatic UC and in phase III randomized trials. Further detailed biomarker studies on the basis of the DV treatment or related data of quality of life would help for the precise selection of benefited patients and acknowledge the patients' quality-of-life performance from DV treatment.

In conclusion, this combined analysis demonstrated a consistent efficacy of DV against chemotherapy-refractory mUC with a manageable safety profile.

## Figures and Tables

**FIG A1.**
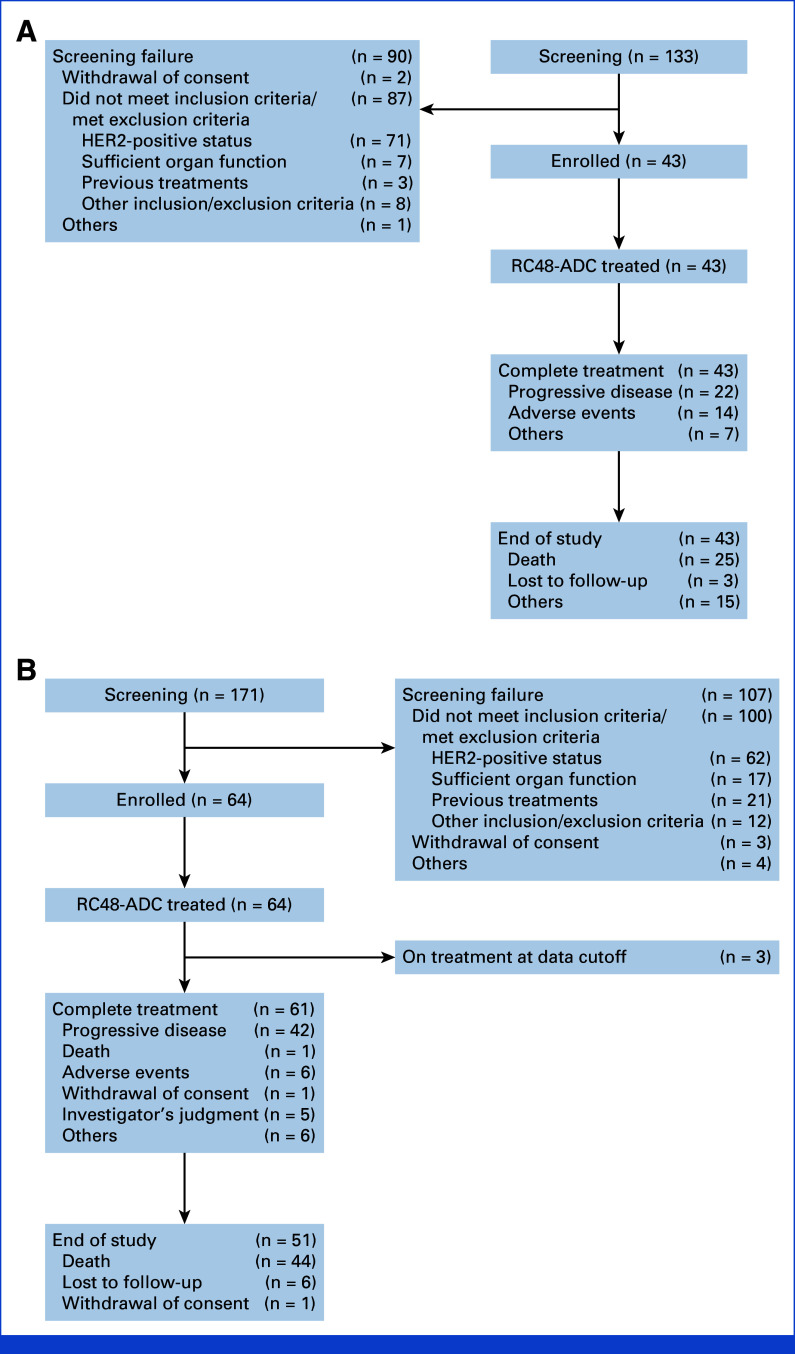
Flowchart of (A) the RC48-C005 trial and (B) the RC48-C009 trial.^a^For the C005 study, two patients had two reasons for screening failure. ^b^For the C009 study, 12 patients had two reasons for screening failure. HER2, human epidermal growth factor receptor 2.

## Data Availability

The clinical data of individual subjects analyzed during the current study are available at http://data.remegen.cn/FolderPublish.aspx?code=Bee2b09b848184a1d81727982202513da.

## APPENDIX 1. STATISTICAL ANALYSIS

The statistical analysis methods of the two studies (RC48-C005 and RC48-C009) were consistent in design, and we maintained close communications and received endorsement from National Medical Products Administration (NMPA) in China. The study designs including the statistical methods of both RC48-C005 and RC48-C009 are summarized in Appendix Tables A1 and A2, as well as their differences in the designs.

For each of the studies, the Clopper-Pearson exact binomial test^20^ was conducted to compare the primary end point (objective response rate [ORR]) with the prespecified historical control. The primary end point of ORR was met for each individual study. The same exact binomial tests are also used in pooled analysis for ORR with the historical control rate and rationale as specified in the RC48-C005 trial. Taken the advantage of the pooled data, the estimates of efficacy and safety profiles are more robust and precise than individual ones. The pooled data adequately followed the PICOS (Participants/Patients, Intervention, Comparison, Outcomes and Study Design) principle as the inclusion/exclusion criteria and design of both studies are consistent (Appendix Table A1). The minor difference (described in Appendix Table A2) is reflected on the stringent inclusion criterion of previous treatment in RC48-C009. The pooled analysis should not be affected. All efficacy end points, including ORR, progression-free survival (PFS), duration of response (DoR), and overall survival (OS), were determined for the intent-to-treat (ITT) population, unless otherwise specified. The ITT population includes all patients enrolled. The planned analyses were discussed and agreed by the health authority. Subgroup analyses with pooled data were performed for patients who had received the second-line treatment and those who had not, respectively, because of the noticeable ratio difference between patients who received greater than or equal to two lines of treatment before enrollment in RC48-C005 (32.6%) and RC48-C009 (85.9%).

In the RC48-C005 trial, a sample size of 60 allowed a 97% power and a one-sided α level of .025 to reject the null hypothesis with an expected ORR of 30% of the investigational drug against the ORR of 10% in control and the dropout rate of 10%. Both ORRs (expected and control) were determined after consultation with NMPA on the basis of the reported range globally in 2016-2017^21^ and expert experience. A total of 43 patients were enrolled and followed up in RC48-C005. The study was stopped early because of high efficacy observed in the prespecified interim analysis. A promising ORR of 66.7% and a disease control rate of 93.3% were observed from data of the first 30 patients. The early stopping of RC48-C005 was also agreed by NMPA.

In the RC48-C009 trial, the planned sample size of 60 patients allows a 95% power and a one-sided α level of .025 to detect an ORR of 52% against a control ORR of 30% with a dropout rate of 10%. The 52% ORR is estimated on the basis of partial follow-up data of RC48-005. The control ORR of 30% was determined after communication with NMPA and considered the highest ORR among existing treatment options^22,23^ and the early readouts of the RC48-C005 study. A total of 64 patients were actually enrolled because of the operational reason that the enrollment was conducted in multiple centers in parallel.

Censor rules listed below were applied to the derivation of PFS and DoR: (1) patients with incomplete or no baseline tumor assessments were recorded as censored, where the censoring date was the first dosing date; (2) patients with progression documented between scheduled visits would be recorded as progressed, and the date of progression was the earliest of the target, nontarget, and new tumor assessment dates; (3) patients without progression were censored with the date of last tumor assessment with no documented progression as the date of censoring; and (4) patients who died before first PD assessment or died between adequate assessment visits were recorded as progressed, and the date of progression was calculated by the date of death.

Two sensitivity analyses of mPFS were performed. The first sensitivity analysis censored patients at the date of last tumor assessment with documented nonprogression before the start of alternative treatments. The second one censored patients at the date of last tumor assessment with documented nonprogression before more than one missed visit. The findings from the sensitivity analyses are consistent with the results of the primary analysis of mPFS.

In addition, the median follow-up was calculated as the interval between the first disitamab vedotin treatment and the last known survival visit among patients censored for OS. The safety of the investigational drug was assessed by parameters including adverse events, laboratory tests, and vital signs. Summary statistics (counts and percentages) were reported for the safety end points on the basis of the safety population, which includes patients who received at least one dose of the study drug.

All statistical analyses were conducted using SAS 9.4.

## APPENDIX 2

**TABLE A1.** Detailed Description and the Difference Comparison of the Statistical Designs for RC48-C005 and RC48-C009 Trials

Key Parameter	RC48-C005	RC48-C009	Difference
Study objective	Effectiveness and safety	Effectiveness and safety	NA
Trial design	Single-arm, open-label, multicenter phase II clinical trial	Single-arm, open-label, multicenter phase II clinical trial	NA
Primary efficacy end point	ORR	ORR	NA
Statistical analysis of efficacy evaluation	Exact binomial test using the single-group target value method	Exact binomial test using the single-group target value method	NA
Alpha (α)	.025 (one-sided)	.025 (one-sided)	NA
Power (1–β)	0.97	0.95	On the basis of consultation with the CDE (NMPA)
Target value and rationale for estimation (null proportion, used to estimate sample size)	10% The ORR of second-line immunotherapy ranged from 20% to 30%, and the ORR of chemotherapy under investigation globally in 2016-2017 ranged from 10% to 30%. After consultation with the CDE (NMPA), a target value of 10% was determined	30% At the time of designing C009, a partial follow-up had been completed for C005, in which a rough estimate was made on the basis of the first 30 patients, and it was expected that about 20 patients would achieve an objective response, ORR = 66.7% (20 of 30) Similar to the estimates in C005, the highest ORR among existing treatment options is about 30%. After communication with CDE and considering the good early results of C005, the target value is recommended to be adjusted to 30%	The target value in C005 takes the lower end of the existing drug ORR range (10%). On the basis of the good efficacy data of C005, the upper limit of the ORR range of existing drugs (30%) was selected for the design of C009 All decisions were made after communication with the CED of China NMPA
Expected ORR (alternative proportion, used to estimate sample size)	30% Estimates on the basis of expert experience. Determined after communication with CDE	52% At the time of designing C009, the rough estimate on the basis of the first 30 patients showed that the ORR of 66.7% (20 of 30) can be acquired. The lower-side 5% CI of the ORR is 52%. So in C009, 52% is used as the expected ORR	C005 on the basis of expert experience. C009 is based on the lower 5% CI in the previous follow-up of C005
Sample size calculation results	53	54	On the basis of the above information
Expected dropout rate	10%	10%	NA
Sample size (design)	60 (53% ÷ 90% = 59, approximately 60)	60 (54% ÷ 90% = 60)	NA
Actual sample size	43 Discontinue the study because the effect exceeded expectations	64 Multiple centers were enrolled at the same time. When the study was completed, the actual number of participants exceeded the design number	C005 was terminated early, and C009 exceeded the designed enrollment

Abbreviations: CDE, Center for Drug Evaluation; NA, not applicable; NMPA, National Medical Products Administration; ORR, objective response rate.

**TABLE A2.** The Difference in Inclusion/Exclusion Criteria Between RC48-C005 and RC48-C009 Trials

RC48-C005	RC48-C009
Key inclusion criteria	Key inclusion criteria
Patients with unresectable, locally advanced, or metastatic UC who developed disease progression or intolerance after receiving first-line systemic chemotherapy	Patients with unresectable, locally advanced, or metastatic UC with failure of at least one previous conventional chemotherapy on the basis of gemcitabine, platinum, and taxanes
Age ≥18 and<80 years	Age ≥18 years
At least one measurable lesion on the basis of RECIST 1.1	At least one measurable lesion on the basis of RECIST 1.1
ECOG PS score: 0 or 1	ECOG PS score: 0 or 1
HER2-positive status confirmed by the central laboratory: IHC 2+ or 3+	HER2-positive status confirmed by the central laboratory: IHC 2+ or 3+
Sufficient organ functions	Sufficient organ functions
Key exclusion criteria	Key exclusion criteria
Received treatment with recombinant humanized anti-HER2 monoclonal antibody-MMAE conjugates	Previous treatment with other antibody-conjugated drugs
Known hypersensitivity to recombinant humanized anti-HER2 monoclonal antibody-MMAE conjugate or any of its components	Known allergy to recombinant humanized anti-HER2 monoclonal antibody-MMAE conjugate drug

Abbreviations: ECOG PS, Eastern Cooperative Oncology Group Performance Status; HER2, human epidermal growth factor receptor 2; IHC, immunohistochemistry; MMAE, monomethyl auristatin E; UC, urothelial carcinoma.
